# When public health emergencies hit the front line: a qualitative study of the patient experience in the emergency department and outpatient infectious disease clinic during the 2022 Mpox clade IIb outbreak

**DOI:** 10.1186/s12879-025-11124-w

**Published:** 2025-05-26

**Authors:** Molly Brick, David Rudolph, Gaby Dashler, Amanda Varnauskas, Luxi Qiao, Matthew M. Hamill, Joyce L. Jones, William Garneau, Mustapha Saheed, Gabor Kelen, Kelly Gebo, Bhakti Hansoti

**Affiliations:** 1 Johns Hopkins Department of Emergency Medicine, Baltimore, MD USA; 2 Johns Hopkins Department of Medicine/Division of Infectious Diseases, Baltimore, MD USA; 3 Johns Hopkins Department of Medicine/Division of Hospital Medicine, Baltimore, MD USA

## Abstract

**Background:**

The World Health Organization (WHO) declared mpox clade IIb a Public Health Emergency of International Concern (PHEIC) in July 2022. During the clade IIb epidemic, patients presented to both Emergency Departments (ED) and Infectious Disease (ID) clinics for care. Given the uncertainty and limited knowledge of mpox in 2022, we sought to understand the patient experience to inform service delivery for future outbreaks.

**Methods:**

A retrospective qualitative study enrolled patients who presented to five EDs and one ID clinic in the Johns Hopkins Health System who tested positive for mpox virus (MPXV) between June 1 and December 31, 2022. Patient interviews were conducted in pairs with one researcher as the interviewer and a second as note-taker. A semi-structured interview guide with post-interview coding for content analysis was used to ascertain clinical factors, individual factors, and system factors that impacted the patient experience.

**Results:**

Of 73 patients who tested positive for MPXV, 47 (64.4%) presented to EDs and 26 (35.6%) to the ID clinic; 23 (31.5%) agreed to participate [*n* = 13 ED, 27.7%; *n* = 10 ID, 38.5%]. All identified as male, median age was 36 years [IQR 31–41], 65.2% Black [*n* = 7 ED, 53.8%; *n* = 7 ID, 70.0%], 87.0% men who have sex with men [*n* = 10 ED, 76.9%; *n* = 10 ID, 100%], 69.6% were people living with HIV [*n* = 7 ED, 53.8%; *n* = 9 ID, 90.0%]. Participants diagnosed in EDs perceived difficulties with follow up and isolation, desired more information about tecoviromat, and nearly half reported no contact from local health departments for follow up care or tecoviromat access. Participants presenting to the ID clinic endorsed positive experiences tied to an established and trusted medical home. Stigma, loneliness, and mental health difficulties were consistent themes for all participants.

**Conclusion:**

Patients present to various venues for infectious diseases such as mpox. Many present to EDs as a safety net service or for more severe disease manifestations. Institutional partnerships between ED, ID, and public health authorities, ongoing training, an on-call system for infectious disease threats, de-stigmatization, and focus on successful linkage-to-care pathways are potential ways to improve responses for future novel infectious disease outbreaks.

**Supplementary Information:**

The online version contains supplementary material available at 10.1186/s12879-025-11124-w.

## Introduction

Mpox is a viral disease caused by an Orthopoxvirus (mpox virus (MPXV)) that had historically been confined to the African continent with two distinct clades: Clade I (formerly the Central African clade) and Clade II (formerly the West African clade) [1, 2]. Unprecedented global human-to-human transmission of this zoonotic disease in 2022 prompted the World Health Organization (WHO) to declare mpox clade IIb a Public Health Emergency of International Concern (PHEIC) on July 23rd, 2022 [3]. More recently, on August 14th, 2024, mpox was re-declared a PHEIC for Clade Ib [4]. From January 1st, 2022 to July 31st, 2024, WHO received notification of 103,048 laboratory confirmed cases throughout 121 Member States of each of the 6 WHO regions, including 33,556 cases in the United States [5]. Mpox is transmitted during close human contact including sexual activity [6], causing significant morbidity and mortality in patients who are immunocompromised such as patients living with HIV (PWH). A review of the epidemiology of the 2022 mpox outbreak revealed a disproportionate number of infections in men who have sex with men (MSM) and in PWH [7].

There is a wide range of clinical manifestations of mpox [1, 8]. This disease often begins with a prodrome which can include fevers, chills, generalized weakness, myalgias and lymphadenopathy, followed by a rash which often evolves over several weeks, manifesting as macules, papules, vesicles, and pustules [9]. An international study with 19 participating countries by Mitja et al. demonstrated that PWH comprised 38–50% of those infected with mpox [10]. Out of 382 PWH who were infected with mpox, there was a 28% hospitalization rate and overall mortality rate of 15% [10]. High mortality was particularly correlated with low CD4 counts (27% mortality rate with CD4 < 100) [10]. On the heels of COVID, mpox clade IIb cases rose quickly in the US in 2022–2023 with 58 deaths among the approximately 33,000 documented cases [5, 10]. Patients were scared about long term consequences, how to access treatment options, and the implications of diagnosis for family and friends [11].

Patients in the United States present to a variety of care venues (including Emergency Departments (ED), infectious diseases (ID) clinics and primary health care) for both diagnosis and management [12]. The ED serves as the frontline for life-threatening and emergent conditions, playing a critical role during emerging public health crises and serving patients with disproportionate burden of social determinants of health and higher risk of HIV acquisition [13]. While a large proportion of patients that presented to the ED would likely be candidates for outpatient management, there are severe manifestations of mpox disease leading to acute respiratory distress syndrome, pneumonia, bacteremia, and severe dehydration [7, 14]. Mpox is spread by human-to-human transmission and can be mitigated through effective infection prevention and control measures such as hand hygiene, barrier protection, and masking [8].

While the medical and epidemiological aspects of the disease are well-understood, its social dimensions, including the impact of stigma and discrimination on patients, is less studied. Understanding the patient experience with mpox diagnosis, treatment, and management is an essential piece to curbing the spread of the disease. Furthermore, the re-emergence of pandemic mpox with the clade Ib outbreak in 2024 emphasizes the importance of understanding patient experiences that can be extrapolated to further outbreaks. To address this gap, we conducted a qualitative study, guided by the patient experience framework [15, 16], to describe the experiences of patients presenting for care in two distinct service delivery venues in a healthcare system, each characterized by its own challenges, demands, and organizational structure. The goal was not to compare directly care across the two venues, given fundamental differences in their patient profiles. Our aim was to understand the nuanced patient experiences in each unique setting with the aim of informing a holistic strategy for future emerging and re-emerging infectious disease outbreaks that considers both the medical and social needs of patients.

## Methods

### Study design and participants

A retrospective qualitative study was conducted that explored the experience of patients who tested positive for MPXV and presented to the Johns Hopkins Health System between June 1st and December 31st, 2022. Patients were identified if they had a positive MPXV nucleic-acid amplification test (NAAT) from any of five urban EDs (Johns Hopkins Hospital (JHH), Johns Hopkins Bayview Medical Center, Johns Hopkins Howard County Medical Center, Sibley Memorial Hospital, and Suburban Hospital) or the John G. Bartlett Infectious Diseases (ID) clinic at JHH. Patients were also eligible if they had a positive nucleic acid amplification test (NAAT) at another facility and presented at one of our study sites for care including management of complications. Selection criteria was based on index visit of their mpox diagnosis with either a positive test sent from an ED or the ID clinic. There was no crossover of participants between cohorts.

### Data collection

**Fig. 1:**
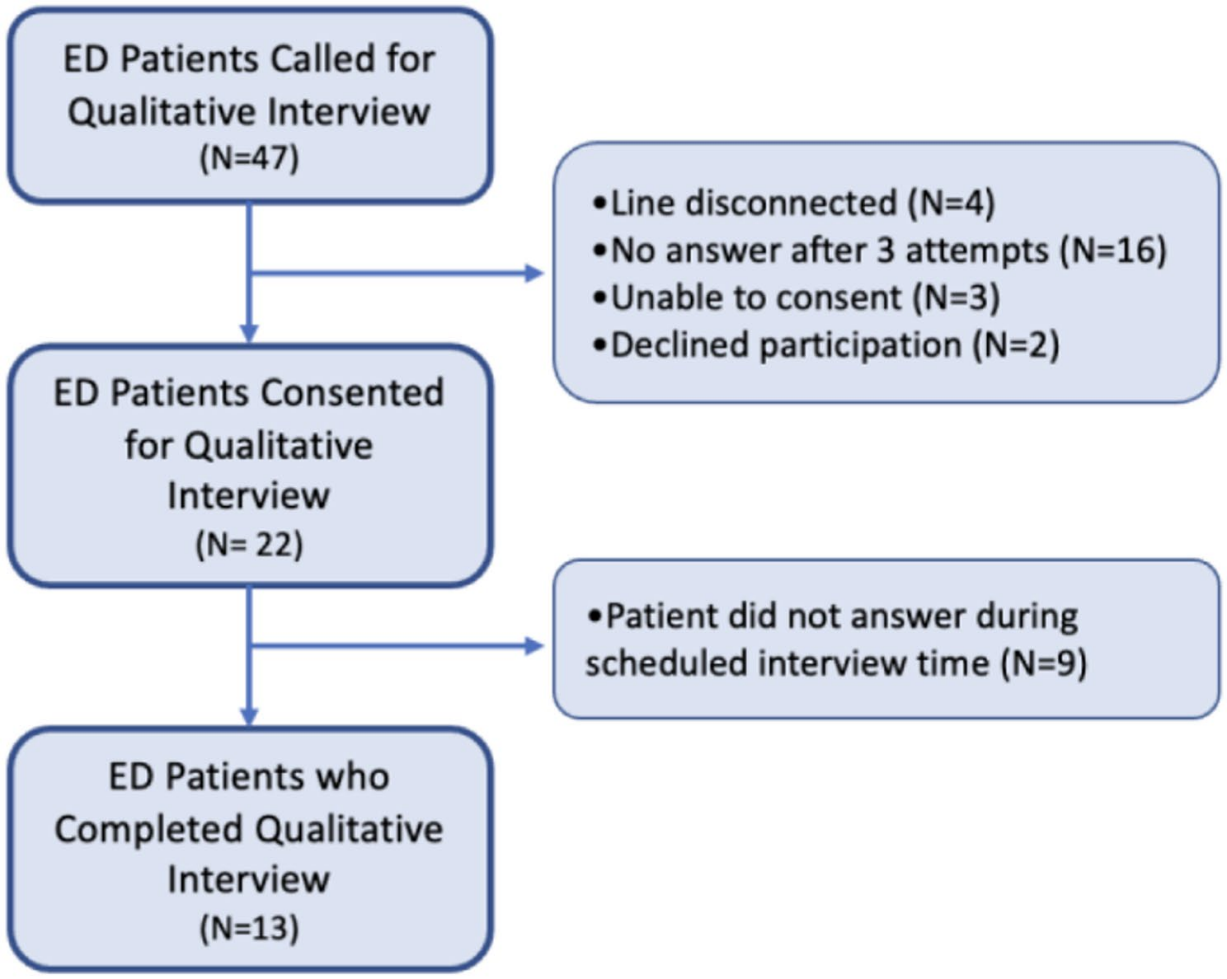
ED Patient Selection Flow Diagram

Participants were identified using an electronic medical record (EMR) data query. The EMR was then screened by the study team for confirmation of mpox diagnosis. Data were extracted into the Research Electronic Data Capture ([REDCap] database, version 13.1.33; Vanderbilt University) [17]. The study team recorded demographic information including age, sex, gender, race, ethnicity, and medical history including HIV status, concurrent illnesses, and behaviors associated with mpox acquisition.

**Fig. 2:**
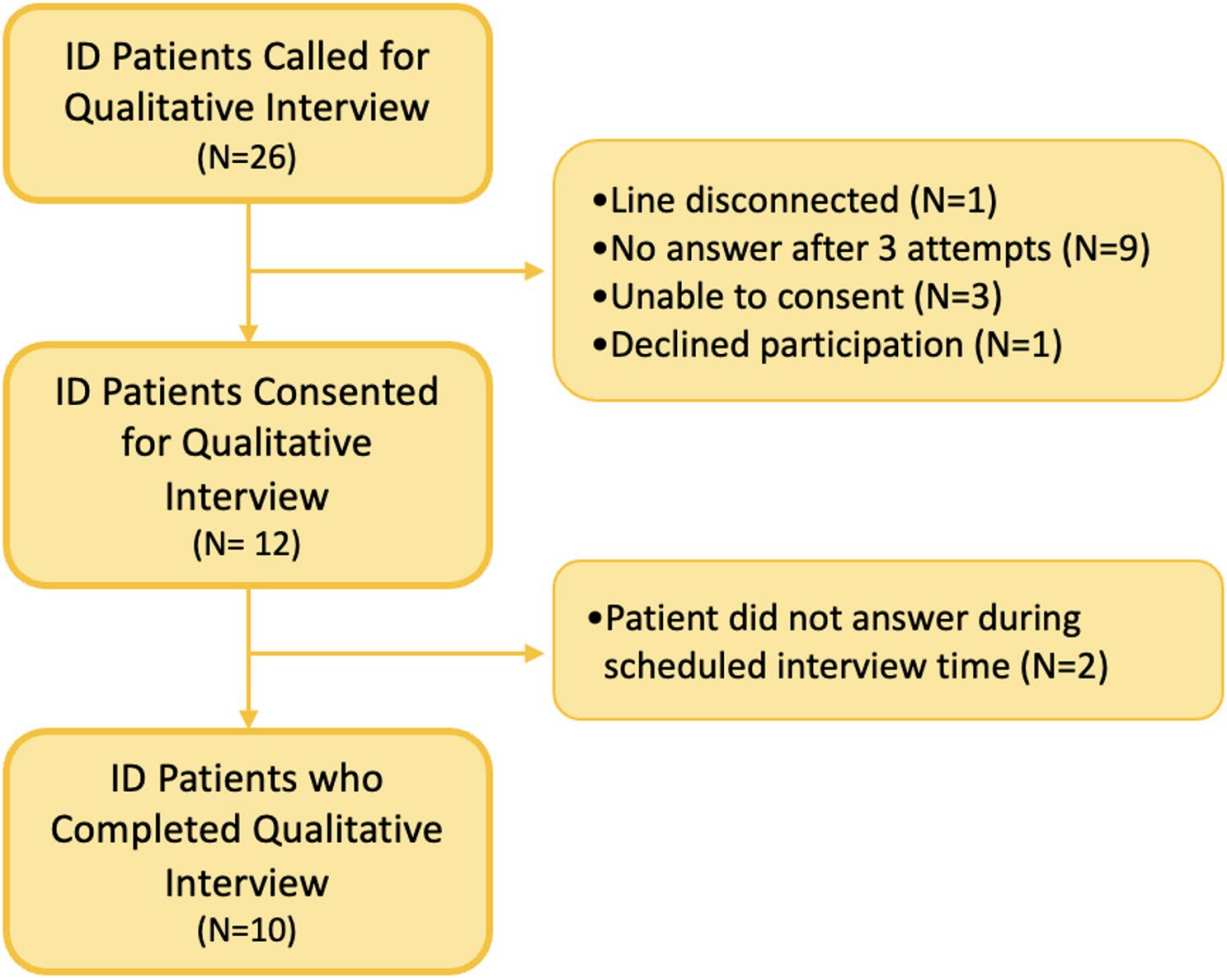
ID Patient Selection Flow Diagram

All participants were then contacted by the study team for a follow up interview after obtaining informed consent. Qualitative data were collected through phone-administered interviews to assess the services and post-discharge care that patients received. Calls were conducted between February 9th, 2023 and September 6th, 2023, approximately 6–12 months after the index visit. Each participant was called up to three times. Attempts were made to schedule calls if participants were willing to be interviewed but not available at the time of the call. Figures 1 and 2 describe the flow of study participants in the ED and ID clinic, respectively.

**Fig. 3:**
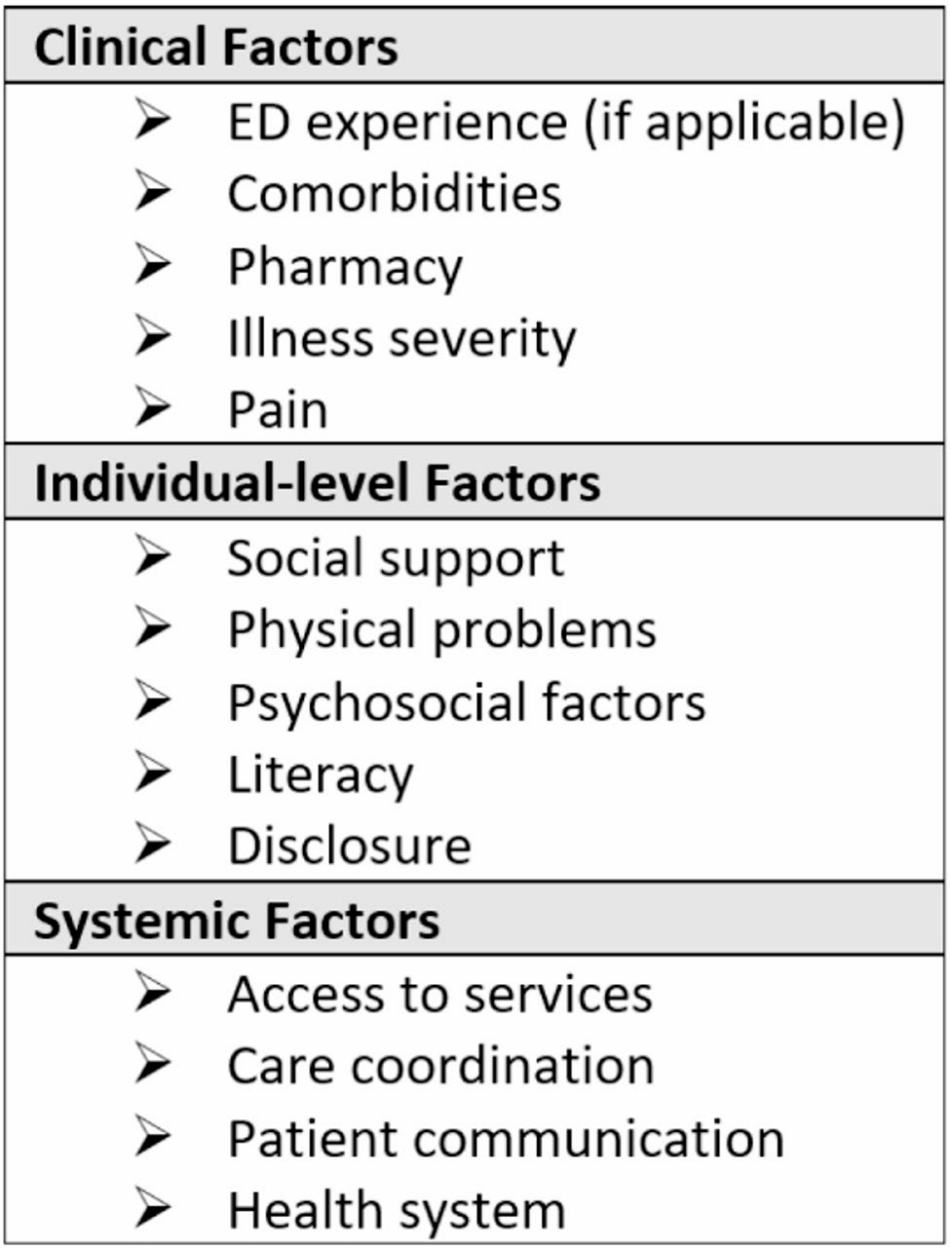
Domains and constructs of the semi-structured interview guide

Semi-structured interviews were conducted in pairs with one researcher administering the interview (interviewer) and another transcribing patient responses (recorder) in real time. The interviewers encompassed four of the authors on this paper (DR, MB, LQ, BH), who are physicians in the Emergency Department. Three of the interviewers (BH, LQ, MB) were female, three (DR, MB, LQ) were resident physicians while BH was faculty in the Department of Emergency Medicine. Recorders were one of three study staff (two females, one male) who had research appointments in the Department of Emergency Medicine. The interview guide was created using a patient experience framework [15, 16], organized into three overarching domains: clinical factors, individual-level factors, and systemic factors impacting patient experience with care. Domains were further broken down into more specific constructs (Fig. 3). Clinical factors focused on the assessment, treatment, diagnosis, and overall management of care for patients. Individual factors explored each patient’s overall well-being, mental health, coping mechanisms, and support systems outside of the clinical setting in response to the diagnosis. Systemic factors assessed access to care and experiences with care coordination and the health system (including interactions with the local institutions and the local departments of health in Baltimore City, Washington D.C., Montgomery County, and Howard County). The full interview guide can be found in the supplementary file (Mpox Qual Semi-Structured Interview Guide).

Interview transcripts were recorded directly into a framework analysis developed using the domains and constructs outlined in the interview guide [18]. Interviews were not confined to a specific time limit and were conducted until thematic saturation, typically lasting approximately 30 min. Patient responses were kept anonymous and recorded into an encrypted, HIPAA-compliant environment to ensure confidentiality and secure data management. Audio recordings were not done due to perceptions of sensitive information such as details about sexual transmission and the prevalence of stigma. The lack of audio recordings or participant transcript review could have affected some of the data quality such as the verbatim accuracy of the transcript. We attempted to mitigate this by asking for clarification and repetition during the interviews and thorough post-interview review by the interviewer and recorder.

### Data analysis

Baseline characteristics were extracted from REDCap to Microsoft Excel and analyzed using simple descriptive statistics.

Qualitative data were analyzed using a framework analysis approach. The framework was developed deductively, using the constructs and domains of the interview guide as themes and sub-themes. All patient responses were transcribed directly into the framework, which consisted of inscribing direct quotes, highlighting key patient experiences, and describing the overall interaction with each patient in real time during the interview. Debriefing occurred immediately following the interviews, where both researchers in the interview pair reviewed the transcripts, supplemented them with additional field notes, and inductively identified and extrapolated additional sub-themes emerging from the interviews using a grounded theory approach [19]. All results were synthesized to summarize the patient care experiences in the different clinical settings.

### Reflexivity statement

All members of the research team are affiliated with either the ED or ID clinic at Johns Hopkins. Our professional backgrounds, personal assumptions, and prior experiences may inadvertently shape our interpretation of patient experiences and our decisions on what aspects of patient care to highlight. We also recognize that power dynamics between the research team (mostly physicians) and patients may influence participant responses during the interviews, particularly given the stigmatizing nature of mpox.

## Results

### Patient demographics

From June 22nd, 2022, to November 16th, 2022, 73 patients were diagnosed with mpox: 47 (64.4%) presented to the ED and 26 (35.6%) to the ID clinic. Of the 73 patients, 23 (31.5%) agreed to participate in an interview [*n* = 13 ED, 27.7%; *n* = 10 ID, 38.5%]. All study participants identified as male, median age was 36 years (IQR 31–41), 65.2% identified as Black [*n* = 7 ED, 53.8%; *n* = 7 ID, 70%], 78.3% as MSM [*n* = 8 ED, 61.5%; *n* = 10 ID, 100%], and 69.6% were living with HIV (PWH) [*n* = 7 ED, 53.8%; *n* = 9 ID, 90%]. Demographics of participants were similar for those who declined interview or were unreachable by phone (Table 1).

**Table 1:** Demographics and clinical presentation of patients with Mpox who participated in the qualitative interviews

	Emergency Department (*N* = 47)		ID Clinic (*N* = 26)	
	Interviewed (*N* = 13)	Not Interviewed (*N* = 34)	Interviewed (*N* = 10)	Not Interviewed (*N* = 16)
*Sex Assigned at Birth*				
Male	13 (100%)	33 (97%)	10 (100%)	15 (94%)
*Gender* Cis				
	13 (100%)	34 (100%)	10 (100%)	15 (94%)
*Age*				
< 30 years	4 (31%)	10 (29%)	1 (10%)	0 (0%)
30–50 years	8 (62%)	21 (62%)	9 (90%)	14 (88%)
> 50 years	1 (7%)	3 (9%)	0 (0%)	1 (6%)
*Race/Ethnicity*				
Black	7 (54%)	20 (59%)	7 (70%)	8 (50%)
White	3 (23%)	8 (24%)	2 (20%)	4 (25%)
Other	3 (23%)	6 (18%)	1 (10%)	4 (25%)
*Insurance Status*				
Private	9 (70%)	14 (41%)	9 (90%)	10 (63%)
Medicaid	2 (15%)	13 (38%)	1 (10%)	4 (25%)
Medicare	0 (0%)	1 (3%)	0 (0%)	0 (0%)
Uninsured	2 (15%)	6 (18%)	0 (0%)	2 (12%)
*Homelessness*				
Yes	0 (0%)	1 (3%)	0 (0%)	0 (0%)
No	13 (100%)	33 (97%)	10 (100%)	10 (100%)
*Injection Drug Use*				
Yes	0 (0%)	1 (3%)	0 (0%)	1 (6%)
No	13 (100%)	33 (97%)	10 (100%)	15 (94%)
*Sexual Behaviors*				
MSM	8 (62%)	28 (82%)	10 (100%)	16 (100%)
*Presenting Signs and Symptoms*				
Rash	12 (92%)	31 (92%)	10 (100%)	16 (100%)
Fever	7 (54%)	16 (47%)	5 (50%)	7 (44%)
Rectal Pain	7 (54%)	3 (9%)	4 (40%)	8 (50%)
Myalgia	1 (8%)	3 (9%)	3 (30%)	2 (13%)
Known Exposure to mpox	5 (38%)	7 (21%)	1 (10%)	2 (13%)
Patients living with HIV	7 (53.8%)	11 (32.4%)	9 (90%)	14 (87.5%)

### Patient experiences

### Clinical factors impacting patient experience with care

#### Treatment availability

Tecovirimat was eventually prescribed to 7 (70%) and 4 (30.8%) patients attending the ID clinic and ED, respectively. Participants attending EDs were more frequently uncertain about their tecovirimat eligibility, while ID patients often knew if they were eligible and how to access tecovirimat. Notably, one ID participant reported having tecovirimat delivered to their home within hours of their clinic visit. ED participants who came with a confirmed diagnosis of mpox requiring hospital admission received tecoviromat in the inpatient setting. All the patients from the ED who were able to be discharged had confirmation of the mpox diagnosis after discharge. Therefore, tecoviromat was only prescribed through referral to the health department or an infectious disease physician.

Most ED participants reported that their pain was very well managed within the ED— “[ED physicians and nurses] made an uncomfortable experience comfortable.” Several also recalled relying on over-the-counter medications to manage their pain after being discharged.

#### Patient interactions with clinical staff

Participants receiving care within the ID clinic reported overall positive interaction, noting professionalism and respect. In certain cases, the interactions with the clinicians helped assuage the participants’ fears, making them feel safe and comforted amidst the feelings of shame and embarrassment that the disease often triggered. ID participants also reported high levels of established trust with their providers and felt confident in the care they received. For example:

“I am living with HIV, but I don’t believe any of that affected anything with my diagnosis or the care I received” – ID participant.

“The ability to see that someone cared – I’ve been to places where you don’t feel wanted or you feel like a castaway” – ID participant.

Moreover, pre-established trust with ID providers appeared to serve as a motivator for seeking care in that location, with one ID participant explaining, “Bartlett is where my doctor is, so I believe in going where my doctor is, instead of going to a place where I don’t know the doctor.”

In contrast, ED participants more commonly reported increased perception of stigma. A few ED participants felt that their experience of mpox was reminiscent of the HIV/AIDS epidemic, feeling as though they had a “label over [their] head,” and that “people think it is the gay disease”.

Additionally, the novelty of this epidemic resulted in a general sense among ED participants that little was known about mpox and how to address it, which resulted in some participants feeling fearful and frustrated.

“I was frustrated at the time; felt like I was a study; I had to suffer so everyone else could get better” - ED participant.

“I felt like people were afraid of me, but I chalked it up to not knowing much about mpox” – ED participant.

“Everyone preaches that you have a disease, but the disease isn’t who you are. I felt like I was mpox, and I was treated that way” – ED participant.

However, despite general feelings of discomfort and stigma, several ED participants felt as though ED staff were competent and knowledgeable in the care they provided. One participant noted that “if I didn’t go to [the ED] there is a possibility I could have passed away,” after having had several negative experiences at other facilities.

### Individual level factors impacting patient experience

#### Psychosocial and physical impact of diagnosis

Both ED and ID participants reported psychosocial difficulties regarding their mpox diagnosis and treatment, expressing feelings of uncertainty and fear. Sentiments such as “Whenever I get a bump, I get a little afraid” (ID participant) and “I was disgusted with myself” (ED participant) highlight the profound emotional toll experienced by individuals grappling with mpox. Several ED participants felt that the diagnosis negatively impacted their mental health, with a few reporting feelings of depression, while others characterized the illness as “mentally challenging.” ID participants with previously diagnosed depression also recalled their symptoms being exacerbated by the diagnosis.

Further exploration of narratives revealed notable concerns related to the physical impact of mpox and how it amplified the associated feelings of shame and embarrassment. One participant from the ID clinic, when describing their experience, explained, “This is not *that bad*, I just don’t like how it looks,” underscoring the nuanced nature of their emotional response to the visible manifestations of the illness. Similarly, an ED participant described feeling as though “[mpox] had changed my body forever.”

#### Social support and disclosure

ID clinic participants denied difficulties with adequate social support, with the majority feeling very comfortable disclosing the diagnosis to loved ones, such as partners, family members, and friends. Furthermore, ID participants did not have any reported issues getting time off work, with the majority already working from home and thus not needing to take significant time off. This greatly facilitated the isolation process, allowing them to maintain their work lifestyle without having to miss work. For others where work from home was not a viable option, they reported no issues getting the necessary time off to isolate.

Among ED participants, the social support they received was varied. Some participants described feeling alone throughout the process; one participant stated that they had no one to help them with their basic needs during isolation. Another participant recalled that “people were incredibly fearful,” further explaining that “people that I hugged for two seconds were screaming, crying, losing themselves.” Some ED participants also reported that they did not disclose their diagnosis to anyone. Like the ID cohort, most ED participants were able to take the necessary time off of work without issues or work virtually during the isolation period. However, one ED participant reported losing their job as a direct result of the long quarantine measures.

### System level factors impacting patient experience with care

#### Care coordination and patient communication

Overall, ID clinic participants recalled positive experiences regarding the duration of their clinical visit. The ED participants generally commented on longer wait times while participants in the ID clinic commented on quick, efficient visits due to having a specific time of the clinic appointment.

#### Follow-up instructions and care

Many ID clinic participants recalled receiving clear follow-up instructions and isolation guidance, and were able to schedule follow up after their index site through the EMR.

For ED participants, however, their experience with follow-up care was more challenging. While some felt satisfied with the level of follow-up they received, some found access to care and follow-up services to be more difficult. Given that many did not have confirmation of an mpox diagnosis at the time of discharge, a common theme from ED participants was confusion on quarantine and isolation instructions. Many ED participants reported relying on the internet or peers to find follow-up information for their care instead of having access to a dedicated primary care provider. One participant in particular described feeling isolated in their follow-up care, explaining that ED staff had trouble finding them a follow-up appointment and “felt like [I] had been left alone to deal with it.” ED participants reported positive experiences when recalling post-discharge provider-initiated follow-up:

One doctor personally called me a week later…was a very good experience, as good as it can be.

After ED visits, participants were instructed to schedule follow-up appointments with their primary care provider or in an ID clinic for linkage to care. Two of the ED participants were scheduled a telehealth appointment from the ED because they did not have a primary care physician.

Reported follow-up from the Department of Health, including advice on ending isolation and partner notification, was inconsistent and lower among participants across both venues. Just under half of the ED participants recalled speaking to the Department of Health; one ID patient recalled receiving follow-up from the Department of Health months after the diagnosis. When there was contact, participants reported a positive experience.

”[The Department of Health] contacted me every two or so days…helped me arrange a follow-up appointment.” — ED participant.

## Discussion

The clade IIb mpox outbreak was unique in that it disproportionately affected patients at higher HIV risk and MSM. However, the latest clade Ib outbreak was concerning for increased human to human transmission and represents the second PHEIC for mpox in just two years [20, 21]. As such, the ED in the United States remains central to the public health response. This is the first mpox study that reflects on the ED patient experience to understand how to strengthen the public health response for future novel emerging infectious disease threats.

In our study, there was perceived stigma from the patient perspective, emphasizing the bias that has affected mpox and past epidemics, most notably HIV. Historically, the language that emerged around the evolving HIV epidemic led to increased mistrust by those affected towards the health care system. For example, physicians and other healthcare providers early in the outbreak frequently referred to HIV/AIDS as a “gay plague” associated with “immoral lifestyle choices” [22, 23]. Similarly, there are numerous accounts of mpox stigmatization by the public labeling mpox as a “gay disease” just like HIV [24, 25].

The impact of this stigma, including a reluctance to access healthcare, will cause delays in diagnosis and spur onward transmission. Strategies to reduce stigmatizing language and behaviors from health care workers include implicit bias training and improving provider clinical knowledge. Nyblade et al. (2009) proposed an intervention that reduced HIV-related healthcare worker stigma by around 50% using participatory training workshops for all hospital staff. These workshops educated healthcare workers on the presence and impact of fear-based and value-based stigma, lessons on HIV transmission, the protection of staff when using universal precautions, and the creation of a policy to create a stigma-free atmosphere [26]. Similar programs for sensitization of healthcare workers, as well as the development of well-designed informational pamphlets or posters to deliver stigma-free information, may also be beneficial for mpox [27].

The service delivery venues in our study differed significantly. ID clinic visits were planned, allowing for pre-established triaging, isolation, and preparedness. There was a smaller group of providers that managed all mpox cases, enhancing patient comfort and trust. The ID clinic organized a specific entrance to a dedicated room that meant patients with suspected mpox could access care without interacting with a busy waiting room. Follow-up care was easily scheduled with the same providers. In contrast, the ED visits could not be pre-planned, handling high volumes and emergent cases, including the tail-end of the COVID-19 pandemic. Patients with mpox, most of which were young and clinically stable, were often triaged to lower acuity, resulting in long wait times due to competing emergencies. Implementation of triage-based ordering or fast-track pathways for infections with rapid sample collection could help reduce ED wait times [28].

Improved access to evolving diagnostic and treatment guidelines for pandemics is essential across all care settings, especially when drug access and prescribing guidance are nuanced. In 2022, tecovirimat was only available through an Extended Access Investigational New Drug (EAIND) where prescribers were required to enroll. In our health system, two-thirds of mpox cases were first diagnosed in the ED, yet tecovirimat prescribing was led by ID physicians. To optimize patient care, a partnership between infectious disease leaders and acute care providers is crucial. Building sustainable relationships fosters the sharing of expert knowledge and standardizes decision-making in uncertain times. For instance, clear guidelines on the latest tecovirimat eligibility criteria, availability, and access could have pre-emptively addressed ED patients’ questions. In addition to protocols reviewed by both ED and ID teams at your institution, we also suggest an on-call ED/ID system to provide timely updates during patient encounters if clinicians have any lingering questions such as nuances in the guidelines for the patient in front of them.

Selection of ED representatives and champions for specific ED populations has been successful in other fields. In Australia, Marsden (2021) described the process of the creation and evaluation of a dedicated nurse-led, physician-championed intervention to better care for geriatric patients whereby a specific ED team focused on comprehensive geriatric assessments and bridged the communication between inpatient and ED teams [29]. Similar successful ED-based interventions have been demonstrated for palliative care [30], intimate partner violence [31], and patients with limited English proficiency for improved use of adequate interpreters [32]. For infectious diseases, a physician champion has been helpful in efforts to improve antimicrobial stewardship practices [33]. When in doubt, healthcare staff unfamiliar with isolation and quarantine, treatment options, and diagnostic considerations can utilize an on-call system to consult with a clinician who better understands the need for timely recommendations to treat all patients equitably and efficiently in overcrowded EDs [34].

Mpox—a clinical condition new to healthcare systems in the US and affecting vulnerable, stigmatized populations—led to uncertainty and fear around quarantine, treatment, follow-up, and prognosis [25]. Communication of test results and guidance about quarantine and isolation post-discharge was challenging, even though it is public health policy to inform patients of their confirmed mpox diagnosis so that they can access appropriate treatment and to prevent transmission to others. Furthermore, the correct public health messaging is important to prevent misinformation. At a minimum, we recommend dedicated call backs for patients with specific guidance on quarantine and isolation measures and updates on the patient’s condition and linkage to outpatient care. Additional case management support may be helpful in the post-discharge communication, ensuring clarity in treatment recommendations and follow up care. An intervention utilizing routine follow up calls for suicidal patients from the ED had a successful mean referral rate of 76%, with 100% referral rates in the presence of an ED champion, further supporting the role of ED/ID champions within the ED staff to bolster engagement in follow up [35].

Linkage to care from the ED for other infectious diseases have shown promise. A 2016 systematic review of ED-based HIV testing programs in the United States showed an overall linkage to care rate of 74.4%; however, this number increased to 80.0% when a patient was in an intensive linkage to care protocol, defined as a physical escort of the patient newly diagnosed with HIV to an outpatient clinic [36]. While physical escort for patients with mpox would not be possible due to universal precautions, virtual telehealth visits could be routinely made prior to discharge of patients presenting to the ED with confirmed or suspected mpox. Automated text messaging systems for patients discharged from the ED have improved linkage to care rates for substance use disorders (70.7% vs. 40.9% in a control group) [37], HIV testing follow up for trauma patients (60.6% vs. 17.9%) [38], and was piloted for adolescent mental health and suicide risk with 75% utilization rates by families when offered text message support for their at-risk children [39]. Assignment of dedicated patient care navigators increased linkage to care for hepatitis C to 43%, higher than other reported linkage to care rates in the literature of 2–10% [40].

Lastly, these patients’ stories shined light on the attention we clinicians must have to social determinants of health. In a study of linkage to care rates for hepatitis C, patients were more likely to be lost to follow up care if they were younger, uninsured, or had comorbid substance use or psychiatric disorders [41]. Interestingly, this study showed opposite trends for linkage to care rates in HIV co-infection and with medical co-morbidities such as congestive heart failure and end-stage renal disease [41], which could be related to existing access to primary care and specialty services. A challenge for linkage to care in vulnerable populations remains when patients do not have access to a telephone, stable housing, and/or are unfamiliar or distrustful of EDs due to the lack of a longitudinal healthcare home for continuity of care [42]. Specific solutions proposed to improve linkage to care in substance use disorder could translate to infectious diseases, including mechanisms for closed loop feedback to ED providers regarding the success of linkage to care for individual patients, creation of a physical space in the ED dedicated to social work engagement or other bridging resources, and provider trainings on how to engage with target populations struggling with linkage to care [42].

### Limitations

There are several limitations to our study. First, while our patient sample was collected from two different care-delivery settings (ID clinic and the ED), these sites are both part of a quaternary care center in a large metropolitan city, and thus hindrances to care may differ for patients from more rural and/or resource-limited contexts. Additionally, resource availability and environment were significantly different in the ED compared to the ID clinic in several identified factors: (1) Tecovirimat was not readily available in the ED; (2) The ED had been relying on local health departments for follow up rather than being already housed within primary care; and (3) Confirmation of the mpox diagnosis was often delayed due to lab turnaround time of greater than 24 h, and therefore were discharged from the ED without a definitive diagnosis. In addition, this study was done during the clade IIb outbreak and therefore findings may not be transferable to clade Ib outbreaks due to differing epidemiology such as affecting more patients in heterosexual sexual networks than the clade IIb outbreak [7].

Our small sample size may limit the transferability of the findings; however, we sought to use detailed questions and probes to achieve significant depth in the data collection process. The small sample size raises the potential concern that those patients who had the most challenging experience when seeking care may be less willing to speak about it. Conversely, patients who had particularly negative experiences could be over-represented because of the desire to provide feedback to improve future care opportunities. Additionally, data was collected at least 6 months after these patients sought care, and thus recall bias may affect the participants’ recollections. While all interviews were conducted under the guidance of the standardized questions listed above, there may be minor variations in diction since interviews were conducted by different team members and thus may influence the answers of the interviewees. Furthermore, there may have been themes we missed if we had started off with an inductive approach to the qualitative interviews rather than the deductive approach with a semi-structured interview guide. Lastly, as with most qualitative data analysis, there is the limitation of subjective interpretation when analyzing the data and therefore must be contextualized within the specific system circumstances.

## Conclusion

Pandemics will continue to bring fear and chaos, exposing the health system gaps that disproportionately affect vulnerable patients. The ED is a safety net, and as the frontline will be a venue where patients with suspected mpox and other emerging or re-emerging infectious threats present for care. Crucial strategies that we advocate to strengthen the public health response from the ED include but are not limited to: (1) Close coordination and communication with infectious disease clinics and local state and public health departments; (2) Designation of an on-call system for novel infectious disease threats; (3) Creation of pathways of care that speed up sample collection; (4) De-stigmatization of service delivery; and (5) Attention to post-discharge linkage to care and follow up.

## Electronic supplementary material

Below is the link to the electronic supplementary material.

Supplementary Material 1

## Data Availability

Focused coding results and data are provided within the manuscript or supplementary information files. Raw data from the interviews themselves are not made publicly available in accordance with privacy laws and patient confidentiality for providing us with their experiences that would otherwise be identifiable due to the unique stories and clinical circumstances.

## Abbreviations

WHO: World Health Organziation
PHEIC: Public Health Emergency of International Concern
ED: Emergency Department
ID: Infectious Diseases
MPXV: mpox virus
PWH: people living with HIV
MSM: men who have sex with men
NAAT: nucleic acid amplification test
EMR: electronic medical record
REDCap: Research Electronic Data Capture
EAIND: Extended Access Investigational New Drug
