# Supplementary material for: When public health emergencies hit the front line: a qualitative study of the patient experience in the emergency department and outpatient infectious disease clinic during the 2022 Mpox clade IIb outbreak
Source: BMC Infect Dis. 2025 May 26;25:754. doi: 10.1186/s12879-025-11124-w (PMC12105172; doi:10.1186/s12879-025-11124-w)
Supplement: Supplementary file 1 — Supplementary Material 1 [file 12879_2025_11124_MOESM1_ESM.docx]

| **Topic** | **Questions** | **Probes** |
| --- | --- | --- |
| System Factors |  |  |
| Access to services | Were you able to arrange follow up services and where? | Where did you get the information? Did the department of health reach out to you?    How was your follow up with your monkeypox diagnosis? How long was it after your visit did you get follow up? |
| Did you need to return for care? | If so, why? | What other options did you try? |
| Care coordination | Did you get support post discharge from the ED/clinic? If so, who? |  |
| Patient communication | Were you able to communicate your diagnosis to household members? Did you communicate the isolation requirements? |  |
| Health systems | Was it clear to you where you could get information from? |  |
| Clinical Factors |  |  |
| ED experience (If applicable) | How was your experience in the ED? | Was it efficient? Did you feel confident? Have your questions been answered? Do you feel like your doctor or other members of the care team communicated clearly and timely about your diagnosis? |
|  | How long did you stay in the ED? | Was this more or less than you expected? |
| Comorbidities | Do you have any other illnesses that impact your ability to recover from MPX? Were these addressed during your ED visit/discharge? |  |
| Pharmacy | Were you discharged with medications? What were they? Did you take them? Did you need refills?    Did you receive Tpox? Do you take it all/did you have any problems with it/insurance coverage? |  |
| Illness Severity | How sick would you say you were? | What made you come to the ED vs. urgent care/PCP/ID clinic? |
| Pain | Was your pain managed in the ED/clinic? Was your pain managed at home? | Do you wish you would have been prescribed more pain medication? |
| Patient Factors |  |  |
| Social support | Were you able to stay off work? Isolate? Manage symptoms | Was it clear how long to isolate for? Were you told? Did you have any problems getting work off? |
| Physical problems | Do you have any physical complaints symptoms after your diagnosis? Are you able to get this addressed? |  |
| Psychosocial | How are you able to deal with the new diagnosis? How has your mental health been since being diagnosed? Were you able to receive the help you need? | What part of your daily routine was affected? |
| Literacy | Were you able to understand your diagnosis? Discharge instructions? Isolation needs? Return instructions? |  |
| Disclosure | Did you disclose your diagnosis to others in your household? Were you counseled to do so? | How were you able to get basic needs fulfilled (like groceries) when you were under quarantine? |
